# The effectiveness of Rock and Water in improving students’ socio-emotional adjustment and social safety: study protocol for a randomized controlled trial

**DOI:** 10.1186/s40359-018-0247-y

**Published:** 2018-07-25

**Authors:** E. C. A. Mertens, M. Deković, M. van Londen, E. Reitz

**Affiliations:** Child and Adolescent Studies, Utrecht University, Heidelberglaan 1, 3584 Utrecht, CS Netherlands

**Keywords:** Rock and water, Intervention, Socio-emotional adjustment, Social safety, Effectiveness, Randomized controlled trial

## Abstract

**Background:**

Students following a low education track have an increased risk for developing problem behaviors. Rock and Water is a widespread, but still poorly evaluated, intervention that aims to improve students’ socio-emotional adjustment and social safety. The aims of this study are to evaluate (1) the effectiveness of Rock and Water on socio-emotional adjustment (i.e., psychosocial wellbeing, sexual autonomy, and resilience) and social safety (i.e., perceived social security in the classroom, aggression, and bullying) and to examine (2) moderators and (3) mediators of its effects.

**Methods:**

Schools are randomly assigned into four conditions: ‘Light’ (a core team of teachers is trained), ‘Standard’ (a core team of teachers and the whole school team is trained), ‘Plus’ (a core team of teachers, the whole school team is trained, and parents are involved), or ‘Control condition’ (Care As Usual). We aim to include 180 7th Grade students in each condition (*N* = 720) across all waves. A multi-informant (i.e., students, parents, and teachers) approach is used to assess the outcomes (socio-emotional adjustment and social safety), moderators (student, trainer, and parent characteristics) and mediators (self-control, self-reflection, self-esteem, and emotion regulation). Video-observations will be analyzed in a subsample to study the possible mediating effect of changes in deviant and prosocial communication among students on the effect on social safety.

**Discussion:**

This project will provide information on the effectiveness of (different levels of school and parental involvement in) Rock and Water, which can be used by schools to decide upon the most efficient way to improve the care for the students. We will be able to shed more light on what works for whom and the working mechanisms of Rock and Water.

**Trial registration:**

Dutch Trial Registration number 6554, registered on the 3rd of July 2017. The design of this study was approved by the Ethical Committee of the Faculty of Social and Behavioral Sciences of Utrecht University (FETC17–015). This study is financially supported by a grant from The Netherlands Organization for Health Research and Development, grant number 531001106.

**Electronic supplementary material:**

The online version of this article (10.1186/s40359-018-0247-y) contains supplementary material, which is available to authorized users.

## Background

In the Netherlands, secondary education (starting at age 12) consists of three education tracks: Preparatory vocational education track, preparatory college track, and preparatory university track. These different education tracks are attended by respectively 43, 28, and 29% of the total student population of secondary education [1]. Students following the preparatory vocational education track (prevocational students) show less autonomy, less academic performance, and less school motivation and commitment than students in the other two tracks [2]. They have an increased risk for psychological problems, such as substance abuse and early sexual intercourse [3, 4], compared to students following the other two tracks. For instance, of the prevocational students about 17% smokes, 48% binge-drinks, and 25% has sex under the age of 17, whereas of students following the preparatory college or university track respectively 5 and 2% smokes, 37 and 25% binge-drinks, and 12 and 6% has sex under the age of 17 [4]. Due to the high level of problems prevocational students might encounter, it is important to positively stimulate their development. Nowadays schools often implement various programs to obtain such aims, especially since the government requires schools to execute a policy to improve students’ socio-emotional adjustment and social safety within the schools.

Rock and Water (R&W) [5, 6] is one of such programs. It is a universal school-based intervention that aims to improve students’ socio-emotional adjustment and social safety by increasing their self-control, self-reflection, self-esteem, emotion regulation and communication skills. R&W uses a psychophysical approach, that is play and exercises are used to increase the strength of youth, to teach them to make (physical) contact with others and to explore, respect and set own and other’s boundaries. The name of the intervention is based on the symbolic principles of ‘rock’ and ‘water’. Rock indicates a rigid and uncompromising attitude: Sticking to your own opinion and not bending for the opinion of others. Water on the other hand represents flexibility and cooperation: Being aware of one’s own opinion, thoughts and feelings and being open to those of others at the same time, willing to cooperate with them. R&W addresses multiple themes including relaxation, self-control, physical and verbal communication, body language, assertiveness, group pressure, sexuality, and sexual violence.

Especially the psychophysical approach makes R&W eminently suitable for prevocational students. These students are less intrinsically motivated for school and (verbal) learning than students of other education tracks [2] and their cognitive abilities are, generally, lower [7]. Learning through play and exercise increases their motivation and is cognitively less demanding than a verbal cognitive approach. Hence, R&W fits in well with the learning style of prevocational students.

R&W is implemented in many countries (e.g., Australia, China, Singapore, France, The Netherlands). Despite this broad implementation, little information about the effectiveness of this intervention is available. Results of several small-scaled studies showed that participants feel more resilient, experience a more positive identity and use more active than passive coping styles after completing R&W [8]. Additionally, a recent study found that boys’ self-reported coercive strategies and verbal manipulation decreased and their self-regulation and general efficacy increased after following the intervention [9].

Notwithstanding these promising results, these studies have several limitations. First, most studies included boys only. It is unknown what the effects are for girls, for youth with different ethnic backgrounds and for prevocational students in specific. Second, only two measurement points were used, prior to the intervention and immediately after the intervention. Changes during the intervention as well as the long-term effects of R&W were not examined. Third, outcomes were narrowly defined [8] which makes it unclear what the effects are on the broader concept of socio-emotional adjustment and social safety. Fourth, often only one informant, mostly the adolescents themselves, participated and only one type of data collection was used, mostly questionnaires. Lastly, the studies did not examine potential moderators or mediators. Hence, no information is available about differential effectiveness for certain subgroups and the working mechanisms of R&W.

To increase the knowledge about the effectiveness and working mechanisms of R&W we will conduct a Randomized Controlled Trial (RCT) in which we plan to overcome the above mentioned limitations. We aim to include prevocational boys and girls from different ethnic backgrounds, conduct measurements prior, during, immediately after R&W and six months later, assess a broad range of outcomes (i.e., socio-emotional adjustment and social safety), use students, parents, and teachers as informants, utilize questionnaires, a computer task, and video-observations, and study potential moderators and mediators.

The first aim of the RCT is to examine the effectiveness of R&W in improving students’ socio-emotional adjustment (i.e., psychosocial wellbeing, sexual autonomy, and resilience) and social safety (i.e., perceived social security in the classroom, aggression, and bullying). We will study R&W in three different conditions and compare it to a control group receiving Care As Usual (CAU; i.e., current school policy to enhance socio-emotional adjustment and social safety of students). We hypothesize that R&W will improve students’ socio-emotional adjustment and social safety and will outperform CAU. The experimental conditions differ in the number of parties that are involved in R&W, that is a core team of teachers, a core team of teachers and the whole school team, and a core team of teachers, the whole school team and parents. The more parties are involved, the broader the ecological focus of the intervention will be. According to the social ecological model of Bronfenbrenner [10] behavior is determined by the interaction of multiple systems (i.e., the individual, family, school). The involvement of multiple systems in the intervention could increase the effectiveness of R&W. Although, this positive effect of involvement of multiple systems is not always found [11]. We will examine if R&W is more effective the more parties are involved, as suggested by Durlak and colleagues [11]. We hypothesize that students’ improvements will be more evident the more parties are involved.

The second aim is to examine potential moderators of the effect of R&W. Several student characteristics will be examined as moderators: Gender, ethnicity, and personality. In the R&W program the emphasis is on physical exercises which might lead to differential effectiveness based on gender and ethnicity. There are differences in the levels of daily physical activity between boys and girls with girls being less physically active [12, 13]. Girls appear to experience higher barriers for physical activities than boys. They are more afraid of being chosen last for a team and of being embarrassed [13]. Therefore, we hypothesize that R&W is more effective for boys.

Also between ethnicities there are differences in the amount and sort of physical activities adolescents engage in. For instance, Black and Asian adolescents are less physically active and show more sedentary behavior than White adolescents [12, 14]. White girls are more likely to be active in sports teams than Hispanic girls whom are more likely to be active in walking for transportation or physical activities at home such as household chores [15]. Kelly and colleagues [15] suggest to tailor physical activity programs based on ethnicity due to differences in factors related to physical activation. For example, White girls appear to have higher levels of self-efficacy related to physical activities which makes them more physically active than Black and Hispanic girls. It might be that White students are more familiar with the sort of physical exercises used in the R&W program than students with other ethnicities. These White students might be less out of their comfort zone due to which they can focus more on the other aspects of the intervention. Thus, we hypothesize that R&W is more effective for White students than for students with other ethnicities.

Previous intervention research has shown that personality can be a moderator of intervention effectiveness. Senf and Liau [16] have found the strongest intervention effects on happiness for individuals with high levels of extraversion and openness and on depressive symptoms for individuals with high levels of extraversion. Asscher and colleagues [17] have shown that Multisystemic Therapy was less effective than treatment as usual in decreasing rule-breaking behavior for adolescent with low levels of agreeableness. Stoltz and colleagues [18] have found the strongest intervention effects on reactive aggression for children with a low level of extraversion and on proactive aggression for children with less extreme levels of conscientiousness. Hence, it is expected that personality also influences the effectiveness of R&W. It is hypothesized that R&W is most effective for students with higher levels of agreeableness and openness, and average levels of conscientiousness. No specific hypothesis concerning extraversion is stated, since the results [16, 18] are inconsistent.

Besides the student characteristics, we will also study trainer characteristics: Gender, ethnicity, education level, level of self-perceived competence, expertise, and degree of training and supervision. Findings concerning characteristics of professional therapists are inconsistent. Whereas some studies have found significant impact of (one of) these characteristics on treatment outcome [19–21], other studies have found no relation [22, 23]. Characteristics of (non-professional) therapists are often neglected in intervention studies. By studying trainer characteristics of non-professional therapists as moderators we will clarify the role of these characteristics on the effectiveness of R&W. We hypothesize that R&W is more effective when trainers are males, have a Western ethnic background, have a higher education level, have more self-perceived competence, have more expertise, and received more training and supervision.

Additionally, we will analyze parental characteristics as moderators: Parental sense of parenting competence and positive parenting (i.e., parental warmth and monitoring). Parents with a high sense of competence feel capable and adequate in interactions with their child [24]. It might be that due to this confidence parents are more susceptible for information about the strategies and ‘language’ of R&W and apply these at home. Positive parenting enhances adolescents’ social competence [25] and the parent-child relationship [26]. It might be that these adolescents feel more competent to incorporate R&W into their daily lives and tell their parents about R&W within that close and trusting relationship. Parents learn more about this intervention which enables them to also apply R&W. Therefore, we hypothesize that R&W is more effective for students with parents with high levels of parental sense of competence and positive parenting.

The third aim is to study the working mechanisms of R&W. Self-control, self-reflection, and self-esteem will be examined as mediators of the effect of R&W on students’ socio-emotional adjustment and social safety. Self-control, self-reflection, and self-esteem are theorized as the three pillars through which R&W aims to establish the desired developments [5]. Additionally, emotion regulation will be examined as mediator. According to the theory behind R&W, students will become better aware of the physical representations of their emotions, for instance muscle tensions and a-rhythmic breathing. Raising students’ emotional awareness is expected to facilitate them to perceive their emotions and regulate them. This improvement in emotion regulation would lead to an increase in their socio-emotional adjustment and social safety [5]. Analyzing these mediators enables us to test the theory of R&W. We hypothesize that R&W will increase students’ self-control, self-reflection, self-esteem, and emotion regulation which, in turn, will enhance their socio-emotional adjustment and social safety.

Furthermore, deviant and prosocial communication (i.e., verbal and non-verbal) will be analyzed as mediators of the effect of R&W on social safety. Communication is a recurrent theme throughout the intervention. It is proposed in the theory that improving the communication of students increases their feelings of social safety, as they learn to show that they care about someone’s feelings, that they are open to others and can become closer to each other [5]. Therefore, we hypothesize that R&W decreases deviant communication and increases prosocial communication which improves students’ social safety.

In sum, in this study we will examine the effectiveness of R&W in improving students’ socio-emotional adjustment and social safety. Moreover, we will examine what works for whom by studying characteristics of students, teachers and parents. Additionally, the working mechanisms of the intervention will be analyzed.

## Methods

### Design

**Fig. 1:**
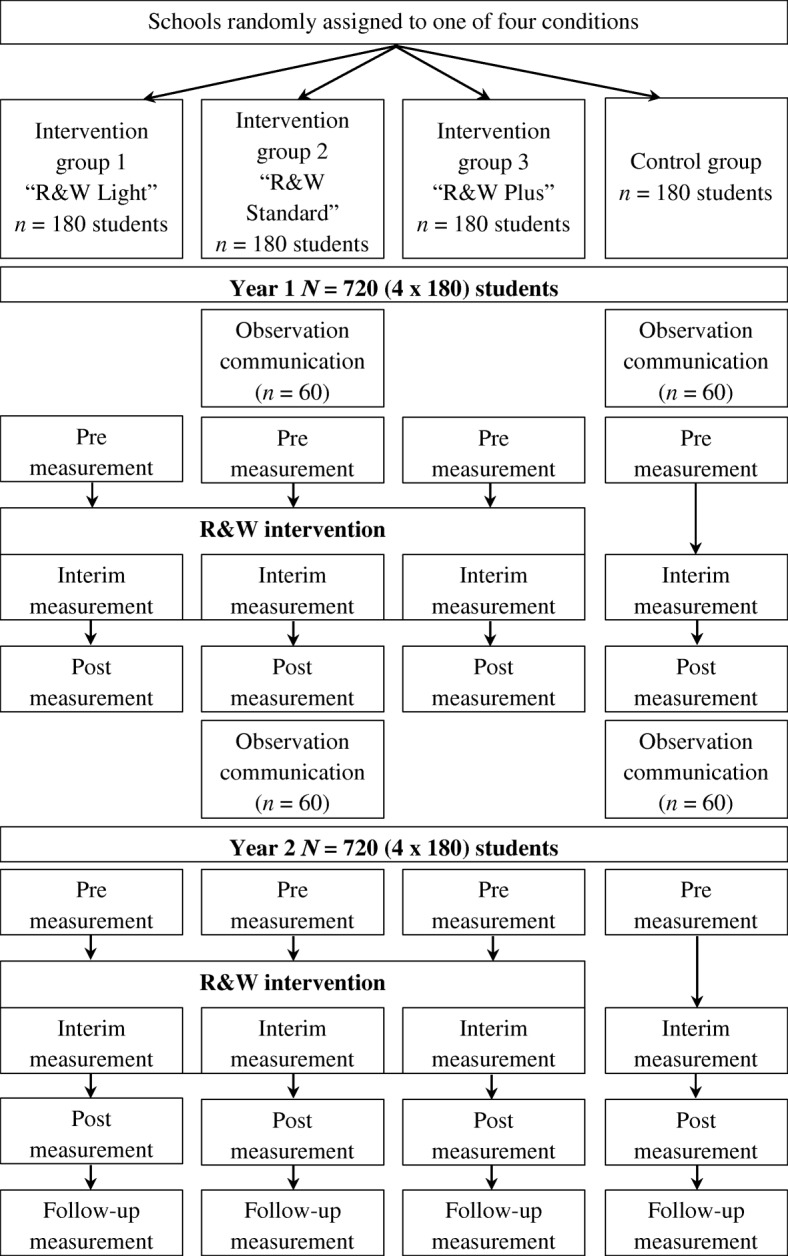
Flow chart

To study the effects of R&W an RCT design is implemented in the 7th Grade of preparatory vocational education level. Schools from different parts of The Netherlands are randomly assigned to one of three intervention groups or to the control group (Fig. 1). In the ‘Light’ condition a core team of teachers is trained with the three-day training course to become certified R&W trainers and implement the R&W program. In the ‘Standard’ condition a core team is trained to become R&W trainers and the rest of the school-team that teaches 7th Grade students follows a three-day training to learn how they can support the R&W trainers of their school and how they can apply R&W in their regular classes. The ‘Plus’ condition is equal to the ‘Standard’ condition with the addition of a parent component. The parents watch a documentary of R&W, get an invitation to join a lesson in the school, receive weekly e-mails with information about the lesson of that week and are stimulated to act on this information, for instance by communicating about R&W or using R&W language. This will not only create a supporting environment within the school, but also in students’ homes. In the control condition students receive CAU; i.e., current school policy to enhance socio-emotional adjustment and social safety of students.

Students receive R&W for two years. The intervention is implemented during physical education classes by (mostly) physical education teachers who followed the three-day training. After successfully terminating this training, these teachers are certified R&W trainers. Socio-emotional adjustment (i.e., psychosocial wellbeing, sexual autonomy, and resilience), social safety (i.e., perceived social security in the classroom, aggression, and bullying), and mediators (i.e., self-control, self-reflection, self-esteem, and emotion regulation) will be assessed at multiple measurement points: Prior, during and after R&W in the first and second year and at follow-up. These concepts are assessed with online questionnaires completed by students, parents, and teachers. The interim measurements (during R&W) are shortened questionnaires and are completed by students after a series of three R&W lessons. Deviant and prosocial communication is assessed in the first year prior and after R&W using video-observations in a subsample. Moderators (i.e., student, teacher, and parent characteristics) are assessed at one time point.

The study is registered with the Dutch Trial Registration (6554) and has been approved by the Ethical Committee of the Faculty of Social and Behavioral Sciences of Utrecht University (FETC17–015).

### Study sample

In total, we aim to include 720 students in this study across all waves, 180 per condition. Participants are students in the 7th Grade, in the second year of the intervention in the 8th Grade, of preparatory vocational education level. Schools are excluded if they currently implement R&W in the whole school or have implemented the intervention in the past two years in the whole school. Additionally, schools for students with special needs are excluded from the study.

### Recruitment

Schools were recruited through the network of the developers of R&W, the Gadaku Institute. An e-mail to certified R&W trainers was sent with information about this study and a message on the private R&W online forum was posted. Trainers asked within their network whether schools that did not implement R&W were interested in participating in this study. When schools were interested they contacted the Gadaku Institute which referred the school to the researchers. The researchers established whether the school was eligible for participation. If the school was eligible, the researcher provided additional information to the schools based on which the school made a decision concerning participation.

Schools were randomly assigned (1:1:1:1 ratio) by stratified block randomization, with blocks of four (i.e., the number of conditions in this study). Schools were stratified by school size (small to moderate sized schools: < 100 students in 7th Grade, large sized schools: > 100 students in 7th Grade) to establish a more equal distribution of students over the four conditions. The randomization numbers were generated by a random number generating computer program.

Students, parents, teachers, and R&W trainers received an information letter to inform them about R&W and the study. Parents also attended a parent-teacher evening where they received additional information about R&W. Students gave active informed consent and parents passive informed consent for participation of the student. Parents gave active informed consent for their own participation in the study. Active informed consent was also acquired for participation of teachers and R&W trainers.

### Conditions

#### Rock and water

**Fig. 2:**
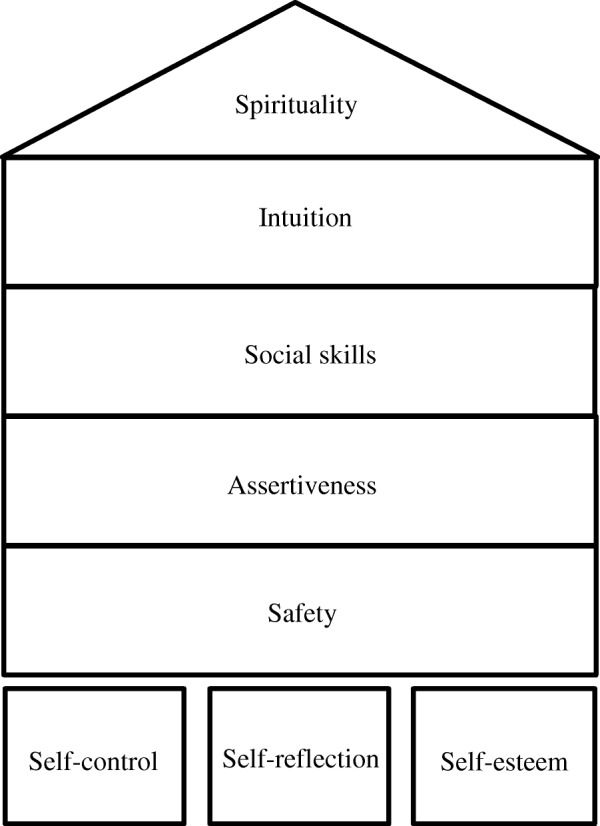
The Rock and Water house

The R&W program is based on the theory of ‘the Rock and Water house’ (Fig. 2). According to this theory there are five levels of the house (themes) that are discussed during the intervention: Safety, assertiveness, social skills, intuition, and spirituality. During the theme safety, feeling safe at home, at school and in society is discussed. Safety is important for students’ development and to find their own way. With the theme assertiveness students learn to deal with difficult situations without losing self-control. The third level social skills emphasizes the importance of communication in our contemporary, multicultural society. Intuition is discussed to make students aware of their preferences and choices, made by intuition, that are determinants for their lives based on their qualities, talents and possibilities. The roof of the house (fifth level) is spirituality. In this theme students learn to follow their own path and gain insight in themselves.

The foundation of the R&W house is built on three pillars: Self-control, self-reflection, and self-esteem. The R&W theory states that acquiring these three skills forms the basis for further development concerning the five themes. By learning to control and direct their energy (self-control), students are able to reflect on their behavior and the consequences (self-reflection). Knowing that they can control, reflect upon and potentially change their actions, students’ self-esteem increases; they know what they are capable of and what they want.

The themes are handled and discussed using a psychophysical approach within a safe, supportive and respectful environment. R&W emphasizes the safe and supportive environment where students are allowed to make mistakes. Such an environment is expected to encourage students’ exploration and learning, addressing personal needs and problems, and establishing positive relations. Respect within the group and between students is created based on the principles and ethics of martial arts on which the physical exercises are based. Students learn basic self-defense skills in which it is supposed that they train their self-control, learn what to do in violent situations and work together respectfully. Additionally, they practice their non-verbal communication by making eye contact and reading the body language of their partner during the exercises.

In the exercises, special attention is paid to students’ body awareness and level of arousal. R&W aims to make students aware that physical signs such as tensions in the muscles, a-rhythmic breathing, and increased heartrate are expressions of emotions, stress and feelings. It is expected that during R&W they learn how to actively relax, find the source of the tensions and connect to their self-control and emotion regulation. This is explained using the terms ‘rock’ and ‘water’. ‘Rock’ indicates a physical tense and firm position with low breathing (i.e., Being aware of one’s own opinion, thoughts and feelings and being able to resist pressure from others). ‘Water’ implies a relaxed but alert position with low breathing (i.e., Knowing one’s own opinion, thoughts and feelings and open to those of others). Students are encouraged to experience that the ‘rock’ and ‘water’ attitudes are on a continuum which they can use to regulate their behavior and interactions.

The R&W program is implemented in the schools for two years. The program starts in 7th Grade with 14 weekly lessons of one and a half hour. In 8th Grade students receive 8 weekly lessons of one and a half hour (see Table 1 for an overview of the topics per lesson). The lessons are given to the class as a whole in large spaces such as a gym hall or drama classroom. The trainers are (mostly) physical education teachers of the schools whom have participated in the three-day training course provided by the Gadaku Institute. These trained and certified teachers are monitored and supervised during the study by coaches of the Gadaku Institute. These coaches have at least once a week contact with the R&W trainers in the schools by e-mail, telephone, or face-to-face depending on the needs of the trainers. They answer questions and give advice regarding the R&W program. Additionally, the coaches observe a lesson of the trainer and provide feedback based on that observation.

**Table 1:** Overview of the Topics of the R&W Lessons in Year 1 and in Year 2

Year 1 (7th Grade)		Year 2 (8th Grade)	
Lesson	Topic	Lesson	Topic
1	Standing strong (Relaxation)	1	Refresh skills year 1
2	Standing strong together (Helping each other in confrontation)	2	Breathing (Relaxation)
3	Physical and mental pressure	3	Body language
4	Bullying (Ignoring)	4	Peer pressure and bullying
5	Bullying (Walking away)	5	Peer pressure (Sexual autonomy)
6	Verbal communication	6	Responsibility and making own choices
7	R&W in school	7	Sexual autonomy (Boundaries)
8	Breathing (Relaxation)	8	Positive thinking and visualization
9	Body language		
10	Personal contact		
11	Experiencing, respecting, and setting boundaries		
12	Experiencing, respecting, and setting boundaries		
13	Intuition		
14	Dealing with intimidating group		

During the lessons, the trainers explain, demonstrate and monitor the exercises indicating what behavior is desired and what is not by giving feedback. The skills and techniques learned in the exercises are repeated over the lessons to enable integration and internalization of these skills in students’ thinking and acting. After an exercise, students reflect in the group upon what they have learned from the exercise and how they could integrate this in their daily lives. Each lesson is ended with a physical condition exercise such as push-ups or jumping rope. In the first lessons, students set individual goals for themselves concerning these physical condition exercises. At the end of the R&W lessons, in 7th Grade and in the 8th Grade, they evaluate if their goals are met or not. After each lesson, students complete homework assignments in their workbook. In this workbook a summary of the lesson and some questions are given to the students. This aims to stimulate the transfer of the taught skills to their daily lives and to practice the skills outside of the lessons.

The physical exercises are performed with different partners which is thought to increase the coherence of the group in which the R&W program is implemented. Students learn how to work and play together as a class. During the exercises they have to control their strength, set boundaries and be respectful towards each other. It is expected that learning how to connect and listen to others while being calm and keeping their strength, they improve their social skills and increase the social support within the class.

#### Control condition

Students will receive CAU: Current school policy to enhance socio-emotional adjustment and social safety of students. In The Netherlands, all schools have such a policy, since it is obligatory. However, the operationalization of the policy can vary widely. Some schools have a program or training other than R&W (e.g., lifestyle lessons), other schools have a policy on management level (e.g., anti-bullying contracts with students).

#### Instruments

An overview of the concepts, instruments, measurement points and informants is presented in Additional file 1: Table S2.

#### Socio-emotional adjustment

To assess psychosocial wellbeing students, parents and (non-trainer) teachers complete the short version (12 items; e.g., “I am stubborn.”) of respectively the Youth Self Report [27, 28], the Child Behavioral Checklist [29, 30] and the Teacher’s Report Form [31, 32] based on the study of Chorpita and colleagues [33]. Additionally, students and parents fill in the subscale Psychological wellbeing (7 items; e.g., “Did you had fun?”) of the KIDSCREEN-27 [34].

Sexual autonomy is reported by the students. For this, items from a national study in The Netherlands concerning sexual health [35] are used. These 5 items represent interaction competence concerning control, assertiveness and self-esteem (e.g., “I have little influence on what happens.”).

To measure resilience, students, parents, and (non-trainer) teachers complete the Connor-Davidson Resilience Scale short version [10, 36]. The 10 items reflect the self-beliefs to cope with difficulties in life (e.g., “Able to adapt to change.”).

#### Social safety

Perceived social security in the classroom is measured using the subscales Comfort (4 items; e.g., “In this class, I can be myself.”), Conflict (4 items; e.g., “In this class, children argue with each other.”), and Cohesion (4 items; e.g., “In this class, everyone likes each other.”) of the Classroom Peer Context Questionnaire [37] completed by students and (non-trainer) teachers. It assesses the perception of school culture, for instance how positive, respectful, friendly and helpful students are towards each other and sense of belonging.

Aggression is measured with the Reactive and Proactive Aggression Questionnaire [38, 39]. It assesses reactive (3 items; e.g., “If they tease or threaten me, I get angry.”) as well as proactive (3 items; e.g., “If I do not like a child, I will bully him with others.”) aggression. Students, parents, and (non-trainer) teachers complete this questionnaire.

To measure bullying, students complete the 2 global items of the Olweus Bully/Victim Questionnaire [40]. It measures the frequency of bullying and victimization. Additionally, students complete brief sociometric nominations assessing social acceptance, popularity and classmates’ roles concerning bullying.

#### Moderators

Students’ gender and ethnicity and trainers’ gender, ethnicity, education, expertise, and degree of received training and supervision are assessed with questionnaires developed for this study.

Students’ personality is reported by the student and parent using the Quick Big Five [41]. It consists of 30 items (i.e., characteristics; e.g., nice, sympathetic, organized) on which the informant can indicate to what extent that characteristic suites the participant.

Parental sense of parenting competence is assessed with the subscale Competence of the Parenting Stress Index [42] completed by the parent. It measures the degree to which parents feel they are capable enough and have enough skills to cope with their child. The subscale contains 8 items (e.g., “Raising my child is harder than I expected.”).

Positive parenting will be measured using the subscales Warmth and Monitoring from the Co-parenting Behavior Questionnaire [43] completed by the parent. The subscale Warmth (7 items; e.g., “I spend time doing fun things with my child.”). measures the extent to which parents show parenting behavior to make their children feel comfortable, accepted and approved. The subscale Monitoring (5 items; e.g., “I know my child’s after school activities.”) measures parental awareness of different aspects of the children’s life.

Teacher’s sense of competence will be assessed with the subscale Self-efficacy for management of the Teachers’ sense of self-efficacy [44] completed by the R&W trainer. This subscale measures teachers’ confidence in their skills to effectively manage their classroom. It contains 6 items (e.g., “How much can you do to control disruptive behavior in the classroom?”).

#### Mediators

Self-control is assessed with the Self-Control Scale short version [45] completed by the student. It contains 11 items (e.g., “I wish I had more self-discipline.”). This questionnaire measures students’ ability to change their inner responses, interrupt undesired behavioral impulses and abstain from acting on these tendencies. Additionally, halfway the questionnaire students complete a shortened version (19 items) of a delayed discounting computer task to measure self-control including a ‘catch’ question [46–48]. Students can choose a smaller, immediate reward or a larger, delayed reward (e.g., “Would you prefer to receive €54 today or in 117 days €55?”). To ascertain that the students have read the questions, a catch question is added, similar in form, amount and delay: “Would you prefer to receive €59 today or in 139 days €21?”

Self-reflection will be reported by the students using the Engage in reflection subscale of the Self-Reflection and Insight Scale [49]. It contains 6 items (e.g., “I don’t often think about my thoughts.”).

Self-esteem will be measured with the subscale Global self-perception of the Self-perception Profile [50] reported by the students. This subscale contains 5 items (e.g., “I’m often disappointed in myself.”).

Emotion regulation is measured using the subscales Impulse control (6 items; e.g., “When I’m upset, I feel out of control.”) and Strategies (8 items; e.g., “When I’m upset, I believe that I will remain that way for a long time.”) from the Difficulties in Emotion Regulation Scale [51] completed by students. These subscales measure students’ ability to control their emotional impulses and the regulation strategies they apply.

Deviant and prosocial communication is assessed using video-observations of same-sex dyads of classmates in a subsample of students in the ‘Standard’ and control condition. This observation task is based on the Peer Interaction Task [52]. The dyads plan an activity together, as warm-up, and subsequently discuss 3 situations concerning daily school situations. Each of these 4 segments lasts 5 min. The 20 min interactions are videotaped and coded. Deviant and prosocial communication is coded based on the Conversation Topic Code [53, 54] and communication ratings [55–57].

#### Treatment adherence

To assess treatment adherence, the trainer indicates after a series of three lessons which strategies were used, level of treatment adherence, and whether the lessons were completed. Furthermore, a subsample of lessons will be observed by an expert in R&W to assess treatment adherence, quality of delivery, participants’ engagement, and adaptations. This coding schema is based on Bishop and colleagues [58]. Treatment adherence indicates the level to which the trainer has conducted the lesson as described in the manual (e.g., “Skipped the trainer exercises?”). Quality of delivery is an indication of the general quality of the lesson (e.g., “Are the goals of the lesson met?”). Participants’ engagement indicates the level to which the trainer actively involves the students in the lessons and the extent to which the trainer can activate students physically (i.e., exercises) and mentally (i.e., reflection; e.g., “Do students respond to questions of the trainer?”). Adaptations are clear deviations from the manual. These can be adaptations to the exercises, structure of the exercise, instructions and adding steps to an exercise (e.g., “What percentage of the exercises of the lesson are adapted?”).

#### Statistical analyses

The power calculations are based on the N:q rule for structural equation models [59]. This rule states that for each free parameter (q) 10 to 20 participants (N) are needed. We took the conservative approach by taking 20 participants per free parameter for our power calculations. In our multigroup LGC model there are 9 free parameters per condition, 36 free parameters in total. Thus a total sample of 720 participants is needed for our analysis, 180 participants in each condition. Since not only students can drop out but also classes (about 20 students) and schools (about 60 to 90 students), we will include three to four schools per condition. Missing data will be handled in M*plus*.

In our data, students are nested in classes which are nested in schools. Therefore, we will examine whether there is significant intra-class correlation on one of the levels (i.e., school, and class) and we will calculate the design effect. Each level with a design effect larger than 2.0 will be modeled in the analyses which allows us to correct for the nested data, that is multilevel analyses [60].

The first aim is to examine the effectiveness of R&W in the conditions which differ in the number of parties involved in the intervention. This will be examined using an analysis of covariance (ANCOVA) for the outcomes of socio-emotional adjustment and social safety, in case the design effect is smaller than 2.0. The dependent variables will be the post-measurements after the second year (8th Grade), the independent variables the condition, and the covariates the premeasurements (7th Grade). If needed, due to large design effects, multilevel regression analyses will be used (This also holds for the other aims). Then, we will analyze the trajectories of change in socio-emotional adjustment and social safety during R&W with multigroup Latent Growth Curve (LGC) modeling in M*plus*. We will examine if these trajectories of change differ significantly between the four conditions.

The second aim, the effect of potential moderators on the effectiveness of R&W on socio-emotional adjustment and social safety, will be examined using ANCOVAs for categorical moderators and regression analyses for the continuous moderators. The interaction effects of the concerned outcome measure with the student, trainer or parent characteristics will be added as an interaction term.

The third aim, studying the working mechanisms of R&W, will be examined by analyzing multiple mediators. We will analyze whether the R&W intervention improves students’ self-control, self-reflection, self-esteem and emotion regulation by performing ANCOVAs. Furthermore, we will analyze whether the change in these concepts mediate the relation between R&W and socio-emotional adjustment and social safety through LGC modeling in M*plus*. We will model the mediators as well as the outcome measures in this mediation analysis on the assessments before, during, and after R&W [61]. Furthermore, we will study whether R&W decreases deviant communication and increases prosocial communication. We will analyze if changes in deviant and prosocial communication are mediators of the effect of R&W on social safety. We will model these indirect effects in M*plus* using bootstrapping.

## Discussion

This study protocol presents the design of a study evaluating the effectiveness of R&W in increasing socio-emotional adjustment and social safety in prevocational students. Previous small-scaled research has shown promising results. With this study we try to overcome the limitations of previous studies by incorporating more measurement waves (i.e., prior, during, and after R&W in the first and second year, and at follow-up), and by using a multi-informant (i.e., students, teachers, parents) and multi-method (i.e., questionnaires, computer task, video-observations) approach. We will be in the unique position to not only examine the effectiveness of R&W in different levels of school and parental involvement, but also to study what works for whom, and the working mechanisms of the intervention.

A possible threat to the study that we foresee is reaching and engaging the different informants (i.e., students, parents, teachers). In general, we try to reduce this possible threat by organizing focus groups with participating schools. During these meetings we discuss the feasibility of our plans and ask for suggestions (e.g., "How can we best reach parents?").

In particular, it might be that we ask students too many questions. They could lose their interest and concentration and eventually give answers without reading the questions to finish the questionnaire sooner. We try to diminish this loss of interest and concentration by digitalizing the questionnaire and by adding a computer task halfway the questionnaire. The digital questionnaire enables students to complete the questionnaire on mobile devices which is probably more interesting to the students than filling in the questionnaires on paper. The computer task makes the students feel like they are doing something different than filling in questionnaires, after which they have renewed energy to complete the second half of the questionnaire. Moreover, students can complete the questionnaire during school hours so that it does not cost them additional time.

Parents might feel disengaged from the study and do not complete the questionnaire. We try to minimize this possible threat by emphasizing the importance of it. Furthermore, both consent and the questionnaire are digital. Parents do not have to actively return a consent letter and can complete the questionnaire at home at a time convenient for them. In addition, we will send reminders for completing the questionnaire to parents through the school’s parental communication system. Moreover, we will organize a raffle to motivate the parents to participate.

Teachers might not have enough time to complete the questionnaires during work hours. They have to fill in a questionnaire for each student which takes up a lot of time. We try to reduce this possible threat by asking multiple teachers per school to complete the questionnaires. Multiple teachers can decrease the burden of the questionnaires since they only have to complete the questionnaires for a subgroup of students (e.g., one class). Additionally, we have personal contact with the teachers completing the questionnaires. This enables us to directly approach a teacher from whom we have missing data.

Another possible threat is insufficient or low treatment adherence. Teachers might deviate from the manual or are not able to complete a lesson due to limited time. We try to gain insight in the quality of implementation by asking questions about treatment adherence every three lessons and by observations during a subsample of R&W lessons. This information can be taken into account in the analyses.

The current study offers the opportunity to examine whether the broadly implemented intervention R&W is effective in positively stimulating the socio-emotional adjustment and social safety of prevocational students. Furthermore, a deeper understanding of the intervention can be gained by studying moderators and mediators. When proven effective, the implementation of R&W in prevocational schools can be stimulated. Additionally, possible adjustments to increase the effectiveness of the intervention for certain subgroups (i.e., students characteristics) might be identified.

## Additional file

Additional file 1: **Table S2.** *Overview of Concepts, Instruments, Measurement Waves and Informants. (DOCX 19 kb)*

## Abbreviations

ANCOVA: Analysis of Covariance
CAU: Care as Usual
LGC: Latent Growth Curve
R&W: Rock and Water
RCT: Randomized Controlled Trial
