# Supplementary material for: The effectiveness of Rock and Water in improving students’ socio-emotional adjustment and social safety: study protocol for a randomized controlled trial
Source: BMC Psychol. 2018 Jul 25;6:36. doi: 10.1186/s40359-018-0247-y (PMC6060546; doi:10.1186/s40359-018-0247-y)
Supplement: Supplementary file 1 — Table S2. Overview of Concepts, Instruments, Measurement Waves and Informants. (DOCX 19 kb) [file 40359_2018_247_MOESM1_ESM.docx]

Additional file 1, Table 2

*Overview of Concepts, Instruments, Measurement Waves and Informants*

|  | Concept | | Instrument | | Measurement | | | | | | | Informant | | |
| --- | --- | --- | --- | --- | --- | --- | --- | --- | --- | --- | --- | --- | --- | --- |
|  | |  | |  | Pre1 | Int^1^ | Post1 | Pre2 | Int^1^ | Post2 | Follow-up | St | T | P |
| *Socio-emotional adjustment* | | | |  |  |  |  |  |  |  |  |  |  |  |
|  | Psychosocial wellbeing | | YSR/CBCL/TRF | | X | X | X | X | X | X | X | X | X | X |
|  |  | | KIDSCREEN-27 | | X | X | X | X | X | X | X | X | X | X |
|  | Sexual autonomy | | Items from “sex under 25” | | X | X | X | X | X | X | X | X |  |  |
|  | Resilience | | CD-RISC 10 | | X | X | X | X | X | X | X | X | X | X |
| *Social safety* | | | |  |  |  |  |  |  |  |  |  |  |  |
|  | Perceived social security in classroom | | Classroom Peer Context Questionnaire | | X | X | X | X | X | X | X | X | X |  |
|  | Aggression | | REPRO | | X |  | X | X | X | X | X | X | X | X |
|  | Bullying | | Olweus Bully/Victim Questionnaire | | X |  | X | X |  | X | X | X |  |  |
|  |  | | Sociometric nomination | | X |  | X | X |  | X | X | X |  |  |
| *Moderators* | |  | |  |  |  |  |  |  |  |  |  |  |  |
|  | Gender, ethnicity | | Developed for this study | | X |  |  |  |  |  |  | X | X | X |
|  | Education | | Developed for this study | | X |  |  |  |  |  |  |  | X |  |
|  | Training and supervision | | Developed for this study | |  |  | X |  |  | X |  |  | X |  |
|  | Competence | | Teachers’ sense of self-efficacy | | X |  | X | X |  | X |  |  | X |  |
|  | Expertise | | Developed for this study | |  |  | X |  |  | X |  |  | X |  |
|  | Treatment adherence | | Developed for this study | |  | X |  |  | X |  |  |  | X |  |
|  |  | | Observation^2^ | |  | X |  |  | X |  |  |  |  |  |
|  | Personality | | Quick Big Five_­_^3^ | | X |  |  | X |  |  |  | X |  | X |
|  | Parental sense of competence | | PSI | | X |  |  |  |  |  |  |  |  | X |
|  | Positive parenting | | CBQ | | X |  |  |  |  |  |  |  |  | X |
| *Mediators* | |  | |  |  |  |  |  |  |  |  |  |  |  |
|  | Self-control | | Self-control Scale | | X | X | X | X | X | X | X | X |  |  |
|  |  | | Delayed discounting | | X |  | X | X |  | X | X | X |  |  |
|  | Self-reflection | | SRIS | | X | X | X | X | X | X | X | X |  |  |
|  | Self-esteem | | Self-perception profile | | X | X | X | X | X | X | X | X |  |  |
|  | Emotion regulation | | DERS | | X | X | X | X | X | X | X | X |  |  |
|  | Deviant and prosocial communication | | Adjusted Peer Interaction Task | | X |  | X |  |  |  |  | X |  |  |

*Note.* Pre1 = premeasurement year 1; Int = interim measurements; Post1 = post-measurement year 1; Pre2 = premeasurement year 2; Post2 = post-measurement year 2; St = student; T = teacher (non-trainer or trainer); P = parent. ^1^Interim questionnaires are shortened and only completed by the students. ^2^Observation of treatment adherence will be conducted by an R&W expert. ^3^Parents complete the Quick Big Five at pre1, students at pre2.
